# Increased complexity of mushroom body Kenyon cell subtypes in the brain is associated with behavioral evolution in hymenopteran insects

**DOI:** 10.1038/s41598-017-14174-6

**Published:** 2017-10-23

**Authors:** Satoyo Oya, Hiroki Kohno, Yooichi Kainoh, Masato Ono, Takeo Kubo

**Affiliations:** 1 Department of Biological Sciences, Graduate School of Science, The University of Tokyo, Bunkyo-ku, Tokyo 113-0033 Japan; 2 Faculty of Life and Environmental Sciences, University of Tsukuba, Tsukuba, Ibaraki 305-8572 Japan; 3 Laboratory of Entomology, Graduate School of Agriculture, Tamagawa University, Machida, Tokyo 194-8610 Japan

## Abstract

In insect brains, the mushroom bodies (MBs) are a higher-order center for sensory integration and memory. Honeybee (*Apis mellifera* L.) MBs comprise four Kenyon cell (KC) subtypes: class I large-, middle-, and small-type, and class II KCs, which are distinguished by the size and location of somata, and gene expression profiles. Although these subtypes have only been reported in the honeybee, the time of their acquisition during evolution remains unknown. Here we performed *in situ* hybridization of *tachykinin-related peptide*, which is differentially expressed among KC subtypes in the honeybee MBs, in four hymenopteran species to analyze whether the complexity of KC subtypes is associated with their behavioral traits. Three class I KC subtypes were detected in the MBs of the eusocial hornet *Vespa mandarinia* and the nidificating scoliid wasp *Campsomeris prismatica*, like in *A. mellifera*, whereas only two class I KC subtypes were detected in the parasitic wasp *Ascogaster reticulata*. In contrast, we were unable to detect class I KC subtype in the primitive and phytophagous sawfly *Arge similis*. Our findings suggest that the number of class I KC subtypes increased at least twice – first with the evolution of the parasitic lifestyle and then with the evolution of nidification.

## Introduction

Hymenoptera comprise many species that exhibit various behavioral traits^1^. Members of the most basal lineages are solitary and phytophagous^2^. Subsequently, a novel parasitoid lifestyle mode evolved, giving rise to prosperous groups of parasitic wasps^2^. In general, adult female parasitic wasps lay their eggs in the host, and their larvae grow inside the host by feeding on the host body^2,3^. From one of their lineages arose Aculeata^2,4,5^, the clade to which all of the eusocial Hymenoptera, such as hornets, ants, and honeybees, belong^6^. The neural mechanisms that underlie the variety of behavioral traits of the hymenopteran insects remain largely unknown.

Insect brains comprise several regions, including the mushroom bodies (MBs, a higher-order center), optic lobes (OLs, a center for visual information processing), antennal lobes (ALs, a center for olfactory and mechanosensory information processing), and subesophageal ganglion (SOG, a center for tasting and feeding behaviors). Centuries ago, Dujardin reported that social hymenopteran species possess large MBs^7^, drawing attention to the role of MBs in the development of sociality ever since. The MBs are a higher brain center conserved across almost all arthropods, including insecta^8,9^, that participate in learning and memory in a wide range of insect species^10–17^. In *Drosophila*, genetic studies revealed that some genes involved in cAMP signaling are expressed preferentially in the MBs, and mutants with defects in the function of these genes exhibit impaired learning ability^18–20^. In addition, in some insect species, including the honeybee, the MBs are also involved in sensory integration^21–23^.

Honeybee MBs are paired structures, each of which has two cup-shaped calyces. Somata of the MB intrinsic neurons, termed Kenyon Cells (KCs), are distributed to the outer surface and inside of the MB calyces^8,24^. MB calyces are composed of KC dendrites. The KCs of adult honeybees are categorized into two classes, class I and II, based on the timing of their development, location of their somata, and projection patterns of their dendrites^24–26^. Class I KC development starts in the prepupal stage^27^ and their somata fill the inside of the calyces, while class II KC development begins during the larval stage, and these KCs have characteristic “clawed” dendrites with their somata surrounding the outer surface of the calyces^24–26^. Categorization into these two classes based on similar criteria is also possible in insects more primitive than Hymenoptera, such as cockroaches and crickets, suggesting that KCs are highly conserved components of the MBs^25,26^. In the honeybee, class I KCs are further divided into three subtypes based on the location and size of their somata, and their gene expression^28^, for review, see refs^29,30^. Class I small-type KCs (sKCs) have small somata (5–7 µm) that occupy the innercore of the calyces, while class I large-type KCs (lKCs) have large somata (7–9 µm) that are located at the inner peripheral region of the calyces, and class I middle-type KCs (mKCs) have middle-sized somata that are located between the lKC and sKC somata^28^.

Class I lKCs, mKCs and sKCs have distinct gene expression profiles for review, see refs^29,30^; many genes involved in Ca^2+^-signaling, which participates in learning and memory in various organisms, are preferentially expressed in the lKCs in the honeybee brain^31–35^, implying their roles in learning and memory. Class I sKCs express *hormone receptor-like 38* (*HR38*), whose expression varies with the division of labor of the workers, implying their involvement in the regulation of worker behaviors^36^. Both class I sKCs and some mKCs are active in the brains of workers engaged in foraging, suggesting their roles in visual information-processing during foraging flights^37,38^. Among KCs, mKCs selectively express *middle-type Kenyon cell-preferential arrestin-related protein* (*mKast*)^28^. As the honeybee is the only organism known to have such KC subtypes, these subtypes are assumed to contribute to the regulation of the highly advanced behaviors of the honeybee.

Farris and Schulmeister (2011) reported, based on anatomic comparison, that the elaborate hymenopteran MB structure arose in parallel with acquisition of the parasitoid lifestyle, and proposed that MB elaboration was driven by the cognitive demands of host-finding behavior^39^, rather than sociality as suggested centuries ago^7^. This led us to the question of what behavioral traits are associated with the elaboration of KC subtypes.

We addressed this question by cloning the orthologs of *Tachykinin-related peptide* (*Trp*) identified in the honeybee^40,41^ as a subtype marker gene, and performing expression analyses in the brains of various hymenopteran insects. Tachykinins are neuropeptides approximately 11 amino acids long that are conserved in metazoa and have a wide range of functions^42–47^. Tachykinin mRNA encodes a prepro peptide that contains several mature tachykinin peptides. In the honeybee brain, *Trp* is preferentially expressed in the MBs and in some cells scattered in various brain regions, such as the OLs, ALs, and SOG^41^. In the MB calyces, *Trp* expression is strong in the sKCs, moderate in the lKCs, and scarce in the mKCs^28,41^. Therefore, all three class I KC subtypes can be distinguished by *in situ* hybridization analysis of this one gene.

In the present study, we analyzed four hymenopteran insect species: the solitary and phytophagous sawfly *Arge similis* (Argidae, Tenthredinidae, Phytophagous groups), which belongs to one of the most basal hymenopteran lineages; the parasitic wasp *Ascogaster reticulata* (Braconidae, Ichneumonoidea, Apocrita); the eusocial hornet *Vespa mandarinia* (Vespidae, Aculeata, Apocrita); and the solitary hairy flower wasp *Campsomeris prismatica* (Scolioidea, Aculeata, Apocrita). The species above are listed in order of phylogeny from basal lineages to lineages close to the honeybee. Phylogenetic relationships are based on previous studies^6,39^.

## Materials and Methods

### Animals

Male and female adults of *C. prismatica* were captured at the Hongo campus of the University of Tokyo (Bunkyo-ku, Tokyo, Japan). Worker, queen and male adults of *V. mandarinia* were obtained from a matured nest collected at Bando-shi, Ibaraki Prefecture on 6^th^ November 2016. Male and female adults of *A. reticulata* were from a stock culture at the Laboratory of Applied Entomology and Zoology, University of Tsukuba (Tsukuba, Ibaraki, Japan). The rearing of *A. reticulata* was based on the method described by Kainoh^48^, and that of host *Adoxohyes honmai* on Tamaki^49^. Emerged adults were fed with honey and water in a plastic container (1.8 L) under the laboratory conditions (25 ± 1 °C, 60 ± 10% RH, andL16:D8 photoperiod), and 2- to 3-day-old wasps were used for experiments. Male and female adults of *A. similis* were captured at the Hongo campus of the University of Tokyo. Eggs of *A. similis* were also captured at the Hongo campus of the University of Tokyo and fed azalea leaves at 25 °C with a 16-h light/8-h dark cycle in the laboratory. Adults were collected within 3 and more than10 days after emergence for further analysis.

### Cloning of partial *Trp* orthologs and RNA probes synthesis

Adults of *C. prismatica*, *V. mandarinia*, *A. reticulata*, and *A. similis* were anesthetized on ice, and their heads were dissected with fine scissors. Total RNA extracted from the brains of each species was treated with DNase I Amp Grade (Invitrogen) and reverse-transcribed using SuperScript III (Invitrogen) with random primers. The cDNA was partially amplified by polymerase chain reaction (PCR) using Ex *Taq* (Takara) with primers designed for relatively conserved regions of hymenopteran preproTrp alignment. Nested PCR was performed for *C. prismatica, A. reticulata, and A. similis*. For primer sequences and PCR conditions, see Supplementary Table 1. The PCR products were cloned using a pGEM-T Easy (Promega) vector. The lengths of the cloned sequences were as follows: *C. prismatica Trp*, 462 bp; *V. mandarinia Trp*, 530 bp; *A. reticulata Trp*, 519 bp; *A. similis Trp*, 448 bp; and *A. similis Elf-1 alfa*, 529 bp. PCR was performed to obtain templates for *in vitro* transcription using Takara Ex *Taq* with M13 forward and reverse primers. The digoxigenin (DIG)-labeled sense and antisense RNA probes were synthesized by *in vitro* transcription using a DIG-RNA-labeling Kit (Roche).

### Phylogenetic tree analysis

*Trp* cDNA sequences of hymenopteran insects and of *Drosophila melanogaster*, *Periplaneta americana*, and *Tribolium castaneum*, were obtained from the NCBI genome database. The sequences were aligned with clustalW using MEGA7 software. Based on this alignment, a maximum likelihood tree was constructed as implemented in MEGA7, using the Tamura-Nei model. The initial tree was obtained using the Neighbor-Join and BioNJ algorithms. The bootstrap test was performed with 200 replicates. The tree was rooted using *Periplaneta americana* as the most external out-group.

### *In situ* hybridization

Whole brains were embedded in Tissue-Tek OCT compound (Sakura Finetek Japan) without fixation and immediately frozen. *In situ* hybridization was performed essentially as described previously^50^. For *A. reticulata* samples with *Trp* probes, signal amplification using the TSA biotin system (PerkinElmer) was performed between washing and color development. Nuclei were stained with 4′,6-diamidino-2-phenylindole (DAPI). Images were captured with a light microscope BX-50 (Olympus), merged using GIMP2.8 software (http://www.gimp.org/), and then the brightness and contrast was adjusted using ImageJ 64.

### Cell body size measurement

The merged and adjusted images of V. *mandarinia* and C. *prismatica* were further analyzed with ImageJ 64 software to test if the three regions in the Class I KCs with different *Trp* signal intensity also differ in the respect of cell diameter. The three regions were manually marked. To randomize, a grid was overlaid using a plugin tool. Outlines of the cell somata under the intersections were hand-traced, and the sizes of the enclosed area were measured. Cell diameters were estimated based on the area size by approximating the cell as a true circle. To verify the cell diameter of the three regions differ each other, *F-*tests were performed first to test the equality of the variances. For two populations with the same variance, Student’s *t-*tests were performed. For the others, Welch’s *t-*tests were performed.

### Data Availability

The sequence data generated in this study can be found in the DDBJ databases under the following accession numbers: LC271568 (C. *prismatica*), LC271567 (V. *mandarinia*), LC271570 (A. *reticulata*), LC271569 (A. *similis*).

## Results

### Identification of partial *Trp* nucleotide sequences from four hymenopteran insects

**Figure 1:**
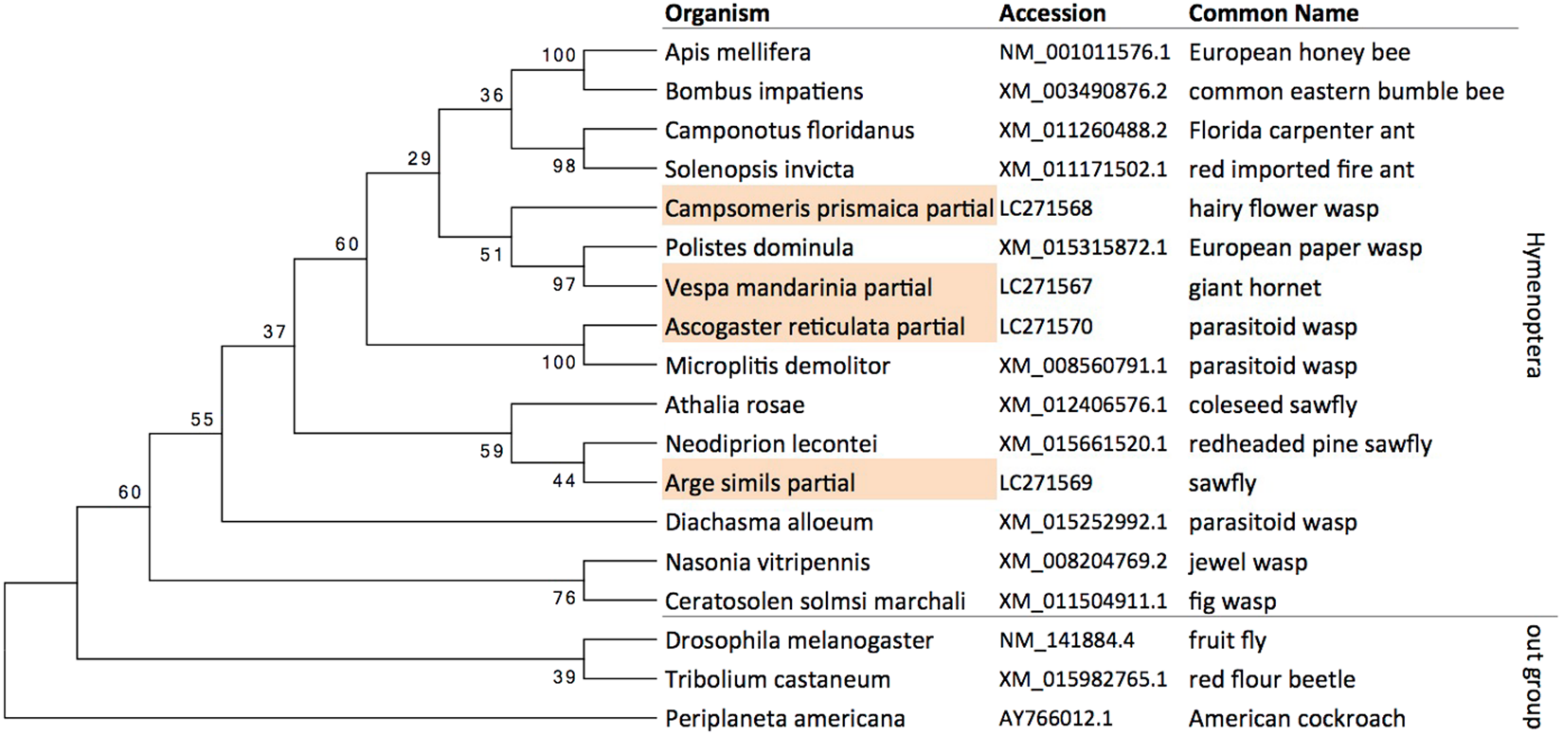
Phylogenetic tree analysis of Trp genes of the hymenopteran insect species. Maximum Likelihood tree of cDNA sequences of tachykinin gene of various hymenopteran insects and others: *Drosophila melanogaster*, *Periplaneta americana, Tribolium castaneum*, and the cloned partial sequences. Bootstrap values of 200 replicates are provided at the nodes. The cloned sequences are indicated with background color orange.

None of the four hymenopteran species we used have a genome database. We first aligned *Trp* nucleotide sequences of various hymenopteran insects with the genome database and designed primers to amplify a region relatively conserved among Hymenoptera. We then obtained partial *Trp* nucleotide sequences by PCR on cDNAs synthesized from total RNAs extracted from the brains of each of the four species. BLAST searches of the cloned sequences resulted in hymenopteran tachykinin genes as the top hits. Next, we compared the similarity of the cloned partial sequences and *Trp* mRNAs of other insects by constructing a phylogenetic tree. All of the cloned sequences belonged to the same group as the other hymenopteran *Trp* (Fig. 1), indicating they are indeed *Trp* orthologs. Based on our findings from genome databases, *Trps* are single-copy in all hymenopteran insects (data not shown).

### Expression analysis of *Trp* in the brain of the solitary wasp *Campsomeris prismatica*

**Figure 2:**
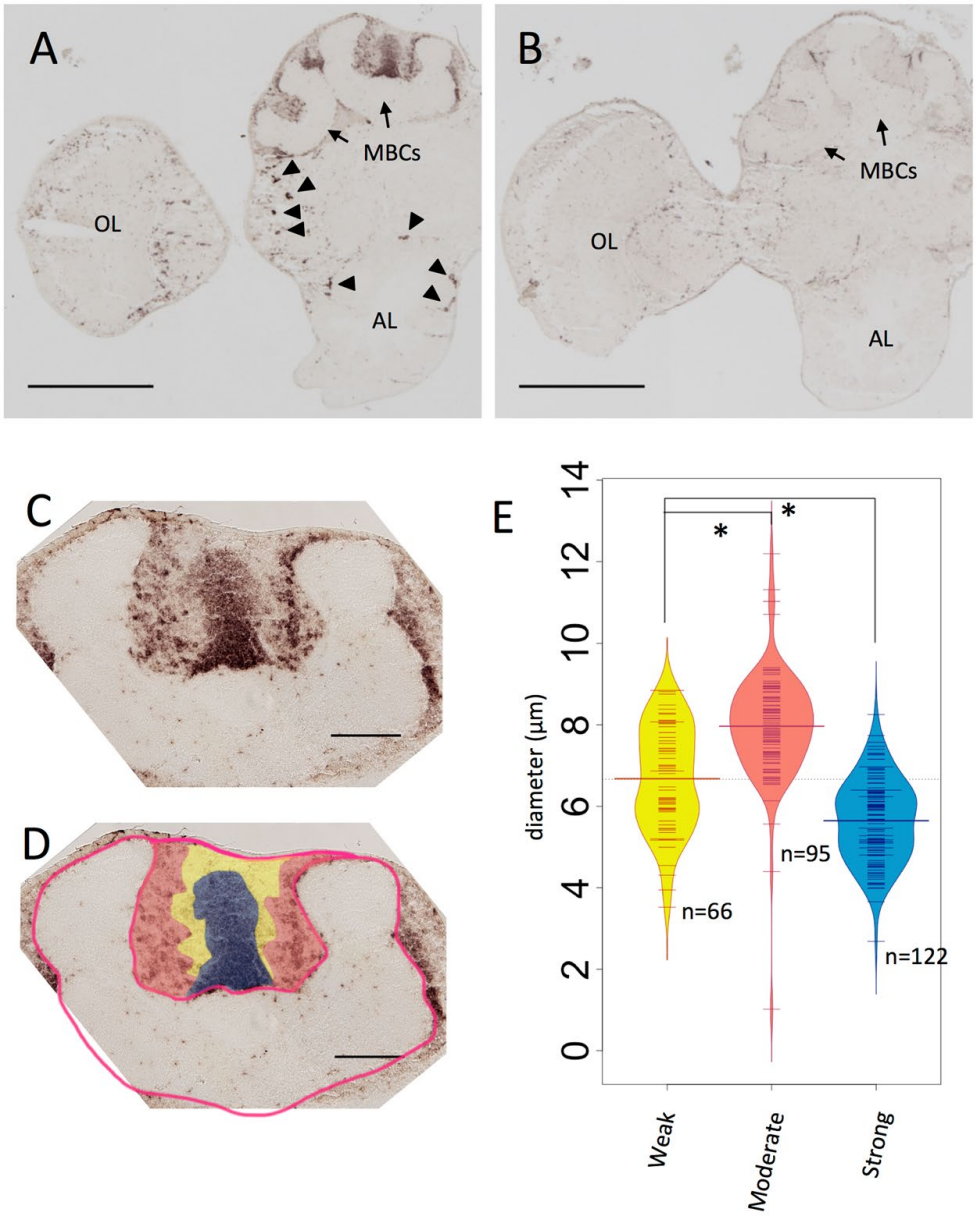
*In situ* hybridization of Trp in hairy flower wasp *C. prismatica* adult female brain. (**A**,**B**) Frontal section of the brain hemisphere hybridized with antisense (**A**) and sense (**B**) *Trp* probes. *Trp* signals scattered outside MBs are marked with arrowheads in (**A**). (**C**) Magnified view of the MB shown in (**A**). (**D**) Illustration indicating the *Trp* signal strength and the calyx is overlaid to (**C**). Kenyon cells were divided into three regions based on *Trp* signal intensity and the location of the somata: the Inner core (with strong *Trp* signal, blue), middle (with weak *Trp* signal, yellow), and inner peripheral (with moderate *Trp* signal, red) regions. The calyx is outlined with red line. (**E**) Distributions of the diameter of the somata of the each region are visualized with beanplot package^60^. Weak, Moderate and Strong correspond to regions colored in yellow, red, and blue in (**D**). MBC, mushroom body calyx; OL, optic lobe; AL, antennal lobe; “n = ”, number of the cells measured; asterisk, p-value < 0.01 (Welch’s *t-*test for Strong-Moderate, Student’s *t-*test for Weak-Moderate). Bars indicate 500 μm in (**A**) and (**B**), 100 μm in (**C**) and (**D**).

Next, we performed *in situ* hybridization analysis using a DIG-labeled RNA probe and 10-μm brain sections of the solitary hairy flower wasp *C. prismatica* (Scolioidea, Aculeata, Apocrita), which is most closely related to the honeybee of the four insect species we analyzed in this study. Strong signals were detected with antisense probe in the MBs (Fig. 2A). In addition, there were scattered signals in some cells in the other brain regions: ALs, OLs (Fig. 2A), and SOG (Fig. S1). In contrast, no signal was detected in sections hybridized with sense probe as a control (Fig. 2B), indicating that these signals represent *Trp* expression. Essentially the same results were obtained for another two female and three male individuals, and no clear difference in *Trp* expression was observed between male and female brains (Fig. S1). The MB-preferential as well as the scattered expression pattern of *Trp* in the other brain regions resembles that of *A. mellifera* ^41^.

The MBs of *C. prismatica* had deep cup-shaped calyces similar to those of *A. mellifera*. Cells with strong *Trp* signal occupied the outer bottom region of the calyces as well as the inner core and inner periphery of the calyces, whereas no significant expression was detected in the regions between the inner periphery and the inner core (Fig. 2C and D). As the location of the somata and the relative *Trp* expression levels of these KCs closely resemble those of the honeybee, it is plausible that they correspond to class II KCs, class I lKCs, sKCs and mKCs, respectively. The somata of honeybee class I subtypes differ in diameter^8,28^. Therefore, we divided the KCs inside of the calyx into three regions based on the intensity of the *Trp* signal (Fig. 2D), and measured their sizes on the image shown. The somata of KCs that probably correspond to class I lKCs, mKCs and sKCs had relatively large, medial, and small diameters, respectively, further suggesting that the four KC subtypes in *C. prismatica* are homologous to those in the honeybee.

### Expression analysis of *Trp* in the brains of eusocial hornet *Vespa mandarinia*

**Figure 3:**
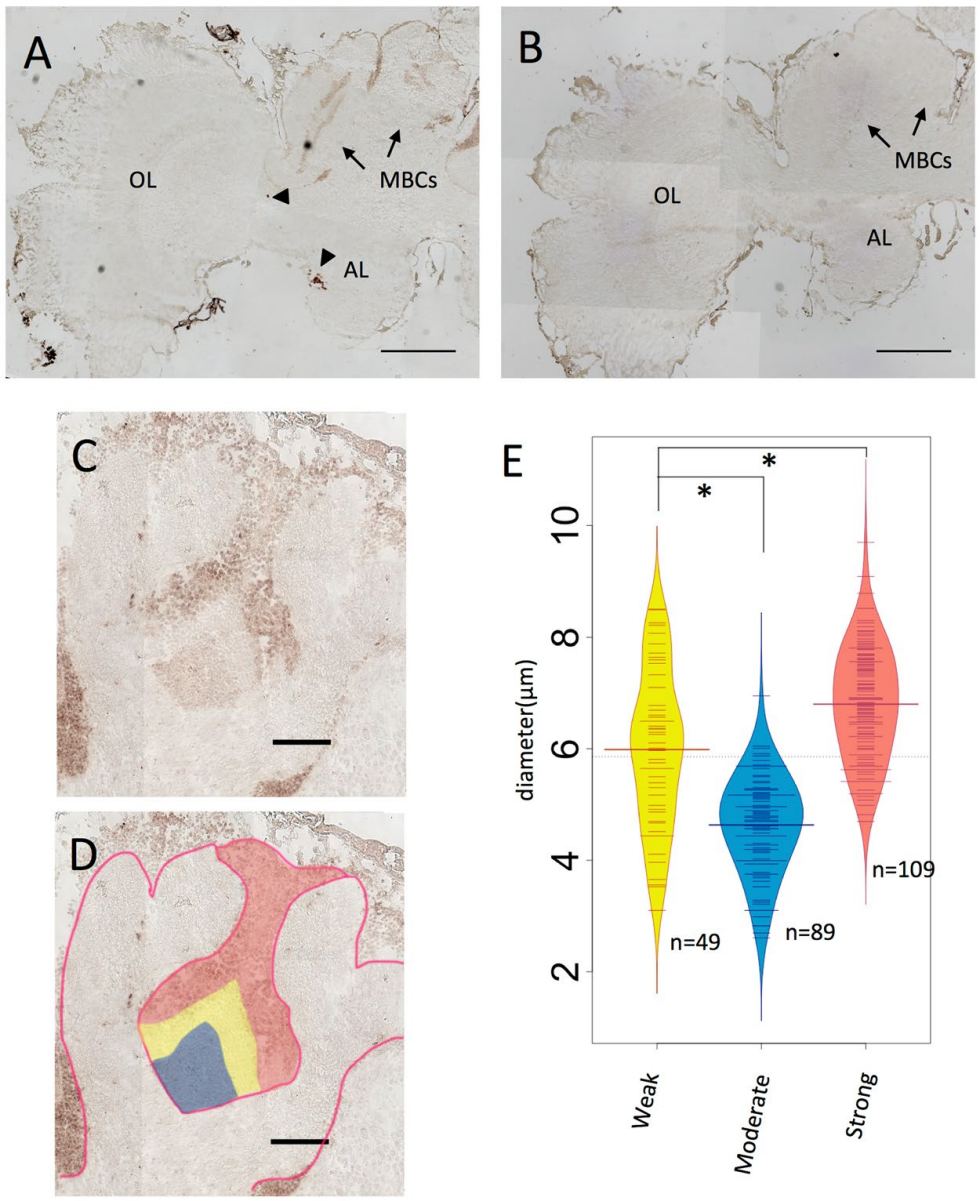
*In situ* hybridization of *Trp* in eusocial hornet *V. mandarinia* adult worker brain. (**A**,**B**) Frontal section of the brain hemisphere hybridized with antisense (**A**) and sense (**B**) *Trp* probes. *Trp* signals scattered outside MBs are marked with arrowheads in (**A**). (**C**) Magnified view of the MB in a serial section of (**A**) and (**B**). (**D**) Illustration indicating the *Trp* signal strength and the calyx is overlaid to (**C**). Kenyon cells were divided into three regions based on the *Trp* signal intensity and the location of the somata: the Inner core (with moderate *Trp* signal, blue), middle (with weak *Trp* signal, yellow), and inner peripheral (with strong *Trp* signal, red) regions. The calyx is outlined with red line. (**E**) Distributions of the diameter of the somata of the each region are visualized with beanplot package^60^. Weak, Moderate and Strong correspond to regions colored in yellow, blue and red in (**D**). MBC, mushroom body calyx; OL, optic lobe; AL, antennal lobe; “n = ”, number of the cells measured; asterisk, p-value < 0.01(Welch’s *t-*test for Moderate-Weak, Student’s *t-*test for Moderate-Strong). Bars indicate 500 μm in (**A**) and (**B**), 100 μm in (**C**) and (**D**).

*In situ* hybridization analysis of the hornet *V. mandarinia* (Vespidae, Aculeata, Apocrita) revealed strong *Trp* expression in the MBs, and scattered expression in the other brain regions (Fig. 3A and B), similar to *C. prismatica* (Fig. 2A and B) and *A. mellifera*. No significant differences in *Trp* expression were observed in the brains of male, worker, and queen hornets (Fig. S2).

The MB calyces resembled those of *C. prismatica* and *A. mellifera* in sense of deep-cup shape, but the well developed lip (subcompartment of the calyx around the rim region) overhanging inward makes their appearance notably different. This structure is consistent with previous observations in another hornet species^51^. Cells with *Trp* signals occupied the outer bottom, inner core, and inner periphery of the calyces, and no signal was detected in the regions between the inner core and inner periphery as in *C. prismatica* and *A. mellifera*, although the signal level of the inner core and inner periphery were reversed (Fig. 3C and D). Essentially the same results were obtained for another worker, queen, and male individuals (Fig. S2). The cells in the inner core (with moderate *Trp* signal), middle (weak), and inner peripheral (strong) regions had relatively small, medial, and large diameters, respectively (Fig. 3E). We assumed that these three cell groups are probably homologous to class I sKCs, mKCs, and lKCs, and the outer bottom cells to class II KCs of *A. mellifera*, based on the similarities in the location and size of the somata despite the reversed relative *Trp* signal level between putative lKCs and sKCs. We also assume that gene expression levels are much more susceptible to the environmental effects and/or individual differences compared to somata location and size^36,40^.

### Expression analysis of *Trp* in the brains of the parasitic wasp *Ascogaster reticulata*

**Figure 4:**
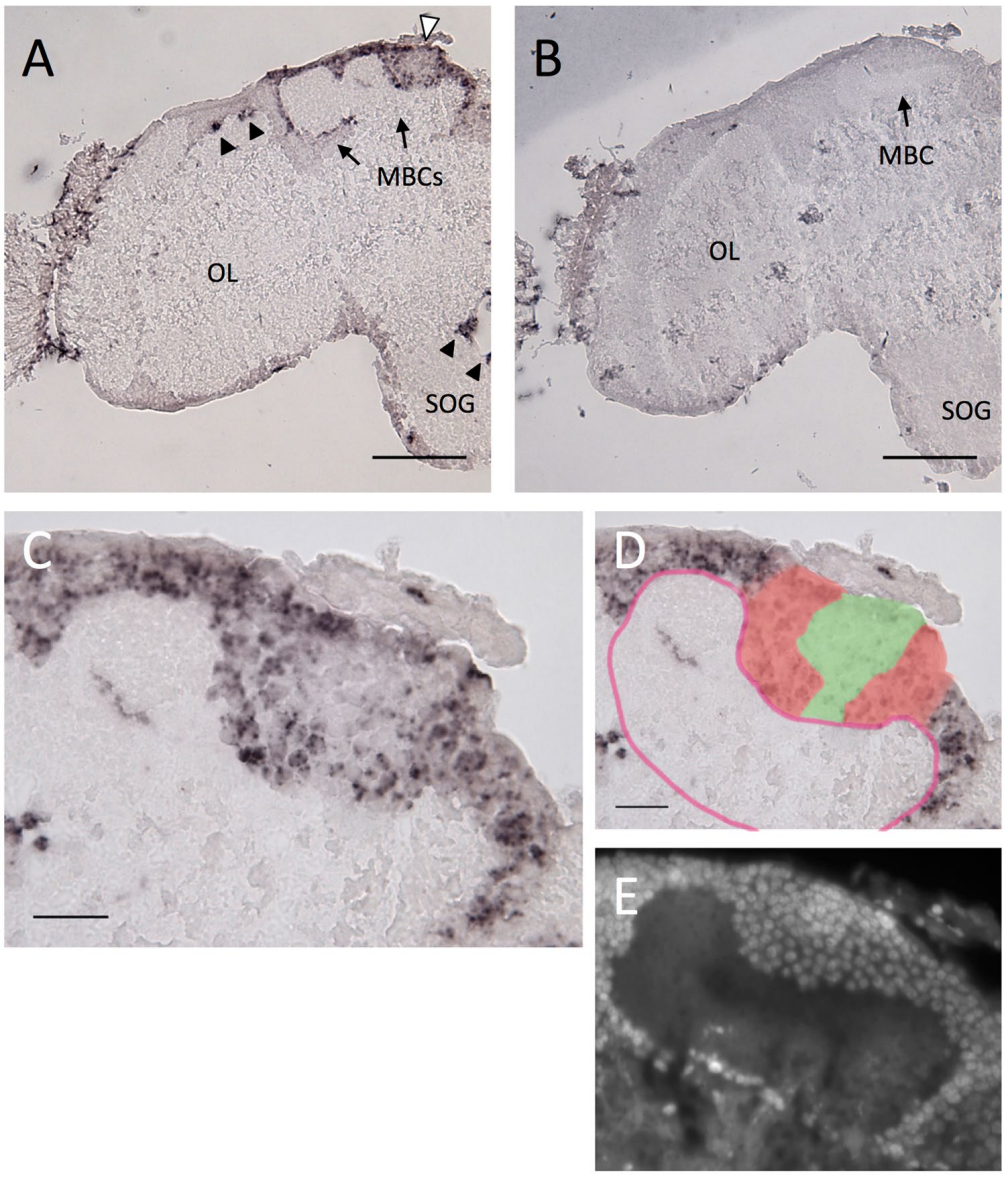
*In situ* hybridization of *Trp* in parasitoid wasp *A. reticulata* adult female brain. (**A**,**B**) Frontal section of the brain hemisphere hybridized with antisense (**A**) and sense (**B**) *Trp* probes. Black arrowheads indicate *Trp* signals scattered outside MBs, and white arrowhead indicates the regions of Kenyon cells inside the calyx with a relatively weak *Trp* signal in (**A**). (**C**) Magnified view of the MB shown in (**A**). (**D**) Illustration indicating the *Trp* signal strength and the calyx is overlaid to (**C**). Regions with strong and weak *Trp* expression are colored with red and green, respectively. The calyx is outlined with red line. (**E**) DAPI staining of a serial section of (**C**,**D**). MBC, mushroom body calyx; OL, optic lobe; SOG, suboesophageal ganglion. Bars indicate 100 μm in (**A**) and (**B**), 20 μm in (**C**) and (**D**).

In the brains of the parasitic wasp *A.reticulata*, we detected strong *Trp* expression in the MBs and scattered *Trp* expression in the other brain regions: in a part of OLs and SOG regions(Fig. 4A and B) and ALs (data not shown), as in *A. mellifera* L^41^, *C. prismatica* (Fig. 2A and B) and *V. mandarinia* (Fig. 3A and B). No significant differences were observed between males and female brains (Fig. S3).

The MBs of *A. reticulata* also had deep cup-shaped calyces similar to those of *A. mellifera* L., *C. prismatica* and *V. mandarinia*, as reported before^39^. This anatomic similarity suggests that the somata that fill the calyx are class I KCs and those that surround the outer surface are class II KCs, as in the other three species. The *Trp* expression in the MBs differed from that in the three species above: cells with *Trp* signals continuously occupied the outside and inner periphery of the calyces, whereas the signal was weak at the inner core (Fig. 4C and D). Measurement of the diameters of these somata proved quite difficult due to vague boundaries of the stained KCs. However, DAPI staining of the serial section demonstrated that while there was no clear difference between the nuclear size of KCs that reside outside and inner periphery of calyces, those of KCs that occupy the inner core of the calyces were more compact and smaller than those of the others (Fig. 4E). Essentially the same results were obtained for another two female and two male individuals (Fig. S3). These observations suggest that although the boundary between class I and II KCs was indistinguishable in this study, there are two class I KC subtypes distinguished on the same basis as the subtypes of the honeybee and the two described species above: the diameter, location of the somata, and *Trp* expression.

### Expression analysis of *Trp* in the brains of the sawfly *Arge similis*

**Figure 5:**
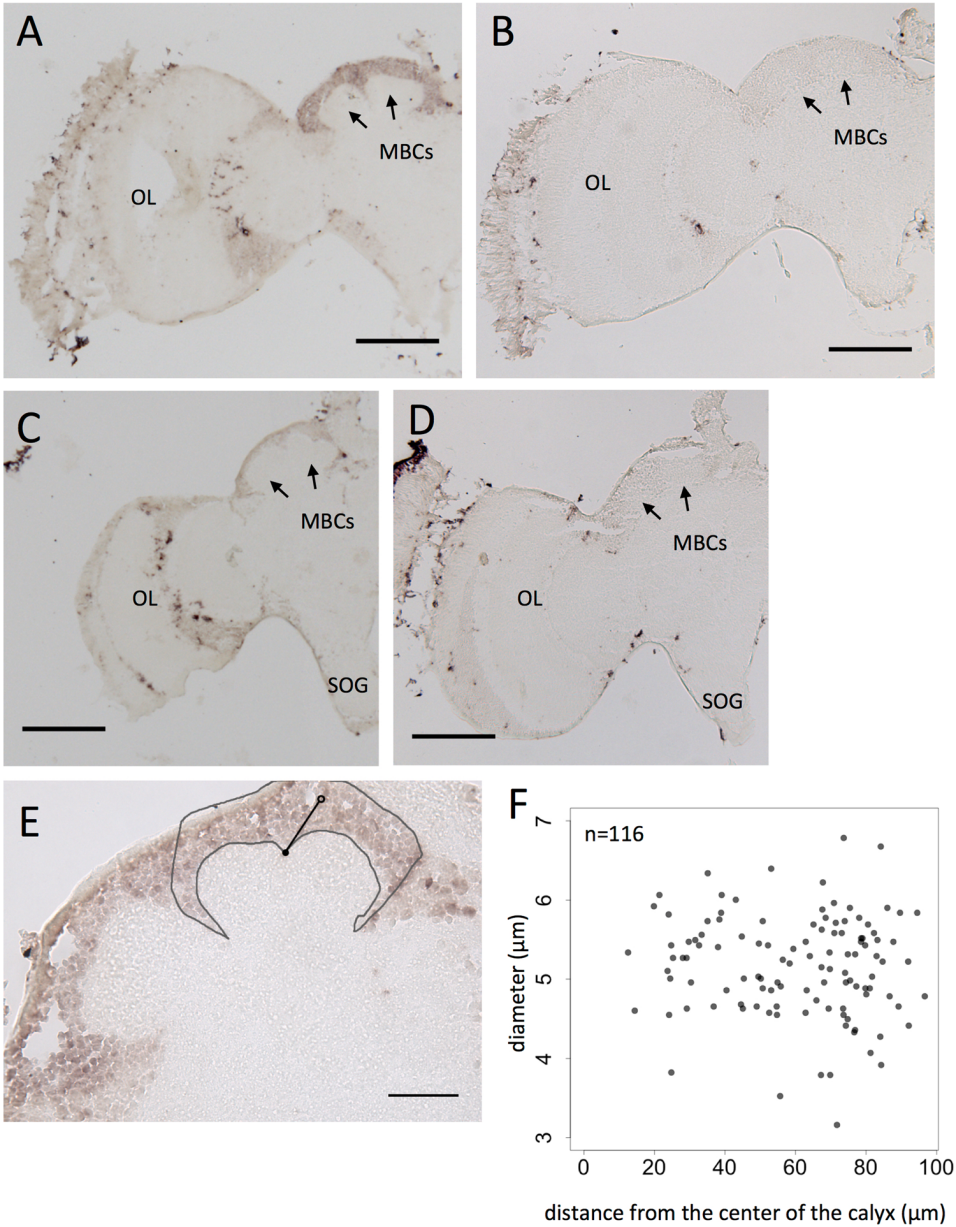
*In situ* hybridization of *Trp* in sawfly *Arge similis* adult female brain. (**A**,**B**,**C**,**D**,) Frontal section of the brain hemisphere hybridized with antisense (**A**,**C**) and sense (B,D) *Trp* probes. (**A**,**B**) An individual younger than three days after emergence. (**C**,**D**) An individual older than ten days after emergence. (**E**) Magnified view of the MB of a serial section of (**A**,**B**). Schematic illustration of the measurements are overlaid. One soma is marked with black circle as an example. Diameter of the somata and the distances (black straight line) from the center of the calyx (black dot) were measured. Somata within the region enclosed with grey line are used for measurements. (**F**) A scattered plot graph of the measurements explained in (**E**). Each dot represents one soma. “n = ”, number of the cells measured; MBC, mushroom body calyx; OL, optic lobe; SOG, suboesophageal ganglion. Bars indicate 200 μm in (**A**,**B**,**C**,**D**), 50 μm in (**E**).

In the brains of the solitary and phytophagous sawfly *Arge similis* (Argidae, Tenthredinoidae, Phytophagous groups), the *Trp* expression patterns differed: some showed stronger *Trp* expression in the MBs than in the other brain regions(Fig. 5A,B), whereas the others showed ubiquitous *Trp* expression throughout the brain cortex (Fig. 5C and D). Stronger *Trp* expression in the MBs was observed in three of five younger (within 3 days after emergence) individuals, whereas ubiquitous *Trp* expression was observed in all four of the older (older than 10 days) individuals (Fig. S4), although whether stronger *Trp* expression in the MBs is unique to the younger individuals is not certain at present. To confirm that the stronger *Trp* signals in some individuals were not due to compacted KCs in their brains, we further performed *in situ* hybridization of the housekeeping gene *Elongation factor-1 alfa* (*EF-1alfa*) on the serial sections of the same brain used for the *in situ* hybridization of *Trp*. Ubiquitous *Ef1-alfa* expression was detected in all seven individuals, both young and old (Fig. S5), confirming that the stronger *Trp* signals in some individuals indeed reflect stronger *Trp* expression.

The MBs had simple ovoid-shape calyces with a small depression at the top, consistent with the previous observation in other sawfly species (Fig. 5A and C)^39^. *Trp* expression in the KCs was ubiquitous in both old and young individuals, and therefore subtypes were indistinguishable in this experiment. We verified the uniformity of the size of the somata by analyzing the correlation between the size and distance of the somata from the center of the depression. There was no significant relationship between them, suggesting that there are no KC subtypes detectable on the basis of the diameter, location of the somata, or *Trp* expression. Albeit undetectable, class I and II KCs are likely to be present in the sawfly because these two classes are assumed to be highly conserved across insecta^25,26^.

## Discussion

**Figure 6:**
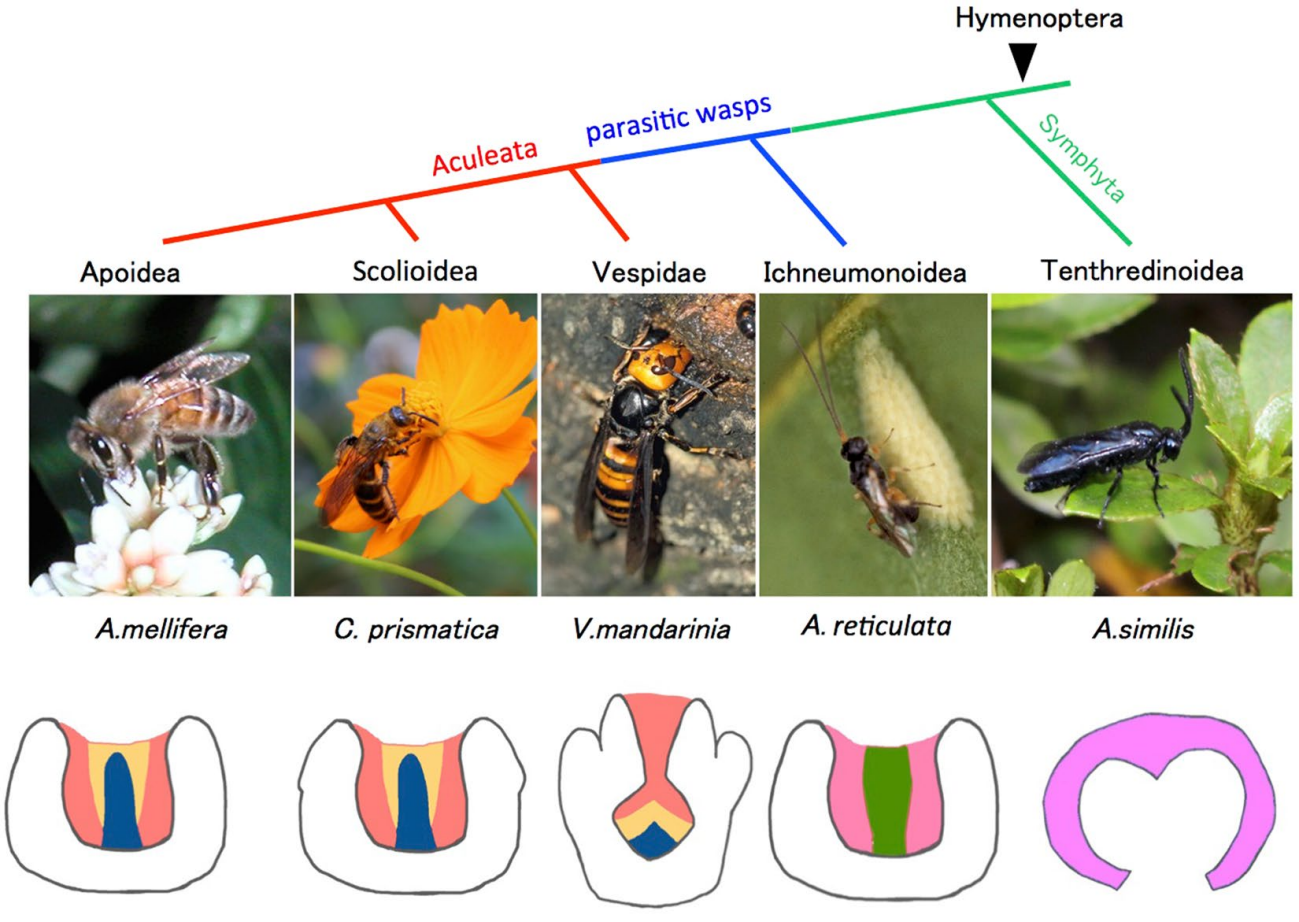
Phylogenic tree and schematic illustration of KC subtypes of hymenopteran species. (Upper panel) Phylogenic tree of honeybee and the four hymenopteran species examined in this study. (Lower panel) Schematic illustration of the KC subtypes. Three Class I KC subtypes that correspond to the lKCs, mKCs and sKCs in the MB are colored with red, yellow and blue for *A. mellifera* L., *C. prismatica* and *V. mandarinia*, respectively, Two putative Class I KC subtypes in the MB of *A. reticulata* are colored with pink and green, respectively. Single putative class I KC subtype in the MB of *A. similis* is colored with pale pink.

Our results demonstrated that KC subtypes became more diverse with the evolution of Hymenoptera: while a member of the basalmost Hymenoptera, *A. similis*, has only one class I subtype; parasitic wasp *A. reticulata* has two; and Aculeata wasps, *V. mandarinia*, *C. prismatica*, and *A. mellifera* have three (Fig. 6). Then what factors had been the background of the acquisition of novel KC subtypes in hymenopteran evolution?

MB calyces commonly receive direct olfactory and mechanosensory inputs from ALs in most insect species^9^. In addition, they also receive direct inputs from OLs in Apocrita and possibly Orussoidea, but not in the phytophagous lineage^39^. The rise of the new subtype in the parasitic wasp might have been triggered by the gain of this novel sensory input into the MB calyx.

Which of the three subtypes of aculeate MBs correspond with class I KCs of A. *similis* and *A. reticulata*? We speculate that class I lKCs of the MBs of aculeate Hymenoptera and class I KCs of *A. similis* are homologous for two reasons. First, class I lKCs of the honeybee receive inputs from ALs^24^ as do the KCs of A. *similis* ^39^. The honeybee MB calyces comprise three subcompartments (neuropils): lip, collar, and basal ring. Olfactory information from the ALs projects to the lip, whereas visual information from the OLs projects to the collar, and both olfactory and visual information project to the basal rings. On the other hand, class I lKCs are likely to have their dendrites in both the lips and collars, whereas class I sKCs have their dendrites in the basal rings^24^. Therefore, the KCs that exclusively receive calycate AL input are lKCs. As for mKCs, their projections have yet to be clarified.

Second, lKCs are assumed to participate in learning and memory because they preferentially express many genes involved in Ca^2+^-signaling^31–35^, for review, see refs^29^ and^30^, which underlies learning and memory in many metazoan species. In insect species more primitive than Hymenoptera, such as cockroaches and locusts, the MBs also function as a center for learning and memory^10,16^. Therefore, KCs present in sawflies presumably resemble the lKCs in honeybee with regard to function. As for the two subtypes present in *A. reticulata*, we think that the cells expressing *Trp* whose somata occupy the inner peripheral region correspond to lKCs, because lKCs likewise occupy the inner peripheral region, express *Trp*, and have relatively large somata. The KCs that do not express *Trp*, which reside in the inner core of the calyces, may correspond to either mKCs or sKCs, or common ancestors of both mKCs and sKCs. This assumption is consistent with the notion that new KCs arose with the direct input of visual information, which mKCs and sKCs are thought to process during foraging flights. Further expression analysis with many other subtype marker genes for review, see refs^29^,^30^ will confirm the homologous relationships between the class I subtypes in honeybees and *A. similis* or *A. reticulata*.

What is the significance of a certain hymenopteran insect acquiring a novel KC subtype? The acquisition of a novel KC subtype may correlate with the development of novel behavioral traits. The sawfly *A. similis*, which has only one class I KC subtype, has a rather simple lifestyle: it lays eggs on the host plant, azalea, and the larvae sustain themselves by eating azalea leaves^52^. The sawfly pupates in the soil under the azalea tree, and the emerged adults live on nectars. This lifestyle does not likely require highly advanced learning and memory ability. In contrast, parasitic wasps, which acquired two KC subtypes and direct input of visual information into the calyces, have extensive learning abilities. They remember the odor, shape, color, and location of the host. *A. reticulata* is also reported to learn plant odor related to the host^53,54^. Consistent with a previous proposal that cognitive demands for host-finding behavior drove the anatomic elaboration of MBs with direct input of visual information to the calyces^39^, the subdivision of KCs may likewise underlie the evolution of parasitic behavior.

The aculeate hymenopteran insects that have three class I KC subtypes, share a behavioral trait to build nests that house their offspring. More precise learning and memory ability is likely required to remember the location and correctly return to their nests. Indeed, aculeate species are known to have sophisticated visual memories^55–58^. We speculate that acquisition of the three KC subtypes with distinct molecular characteristics conferred the aculeate hymenopteran insects the abilities needed for nidification, possibly by functional specialization of each subtype. Considering that all the eusocial Hymenoptera belong to Aculeata as well as a previous report on the possible involvement of the sKCs in the division of labor of workers in the honeybees^36^, it is also plausible that the acquisition of the three KC subtypes is a preadaptation for eusociality.

Lastly, the roles that Trp plays in hymenopteran brains remain unknown. In *Drosophila*, a genetic study demonstrated that Trp promotes male-specific aggression^47^. As Trp is a secretory neuropeptide and thought to affect various neural circuits^42–47^, it is difficult to deduce its role in Hymenoptera. We expect that a combination of the analyses of projection patterns, more detailed molecular characteristics, and functional analysis using genome-editing technology, which was recently established in the honeybee^59^, of each KC subtype will provide important clues to enhance our understanding of the neural bases of spatial memory and social behaviors, and their evolution.

## Electronic supplementary material

Supplementary Information
