# Supplementary material for: Increased complexity of mushroom body Kenyon cell subtypes in the brain is associated with behavioral evolution in hymenopteran insects
Source: Sci Rep. 2017 Oct 23;7:13785. doi: 10.1038/s41598-017-14174-6 (PMC5653845; doi:10.1038/s41598-017-14174-6)
Supplement: Supplementary file 1 — Supplementary Information [file 41598_2017_14174_MOESM1_ESM.pdf]

Supplementary Information

Increased complexity of mushroom body Kenyon cell subtypes in the brain is associated with behavioral evolution in Hymenopteran insects

Satoyo Oya[1], Hiroki Kohno[1], Yooichi Kainoh[2], Masato Ono[3], and Takeo Kubo[1†]

1. Department of Biological Sciences, Graduate School of Science, The University of Tokyo, Bunkyo-ku, Tokyo 113-0033, Japan;
2. Faculty of Life and Environmental Sciences, University of Tsukuba, Tsukuba, Ibaraki 305-8572, Japan;
3. Laboratory of Entomology, Graduate School of Agriculture, Tamagawa University, Machida, Tokyo 194-8610, Japan.

†: Corresponding author:
Graduate School of Science, The University of Tokyo, Bunkyo-ku, Hongo 7-3-1, 113-0033 Tokyo, JAPAN; Tel: +81-3-5841-4446; FAX: +81-3-5841-4447; email: stkubo@bs.s.u-tokyo.ac.jp

Supplementary Table 1. Primer sequences and PCR conditions for cloning of *Trp* and EF1α.

		gene name	TRP				EF1α
		Species	*A.similis*	*A.reticulata*	*C.prismatica*	*V.mandarinia*	*A.similis*
1st PCR	primer	Forward	GGGATTYCAAGGGATGAGGGGRAA	ATGGGTTTYCARGGYATGAG	GGGATTTCAAGGTATGAGAGGG	GGGATTTCAAGGTATGAGAGGG	RATYGGNGGTATYGGAACRG
		Reverse	CCAACGAAYTTRCCKCKWA	YRAATKTACCHCKMAWTTCCC	CMATCGTGGATTYTTYTTWCC	MCGTTTGCCTCTCATACTTTGR	GACGCATRTCRCGNACVGC
	ExTaq(U/50µl)		2.5	2.5	2.5	1.25	1.25
	annealing		48℃	55℃	55℃	55℃	60℃
2nd PCR	primer	Forward	TGGAGGAGGTYATGGASGARA	ATGRGDTWYCARGGRATGCG	ATGGGATTTCARGGAATGAG		
		Reverse	TTCTTTCCCCKCATYCCYTGG	CATTYCTYYTYCCYCTCATYC	CMATCGTGGATTYTTYTTWCC		
	ExTaq(U/50µl)		2.5	2.5	1.75		
	annealing		55℃	55℃	55℃		

All PCR were performed with the reaction mixture of the same composition (ExTaq reaction buffer (plus Mg^{2+}) ×1, dNTPs 0.2 mM each, primers 0.32 µM each), except for the concentration of the polymerase as shown in this table. For all PCR reactions, extension, 72 ℃; denature, 94 ℃; cycles, 30-35.

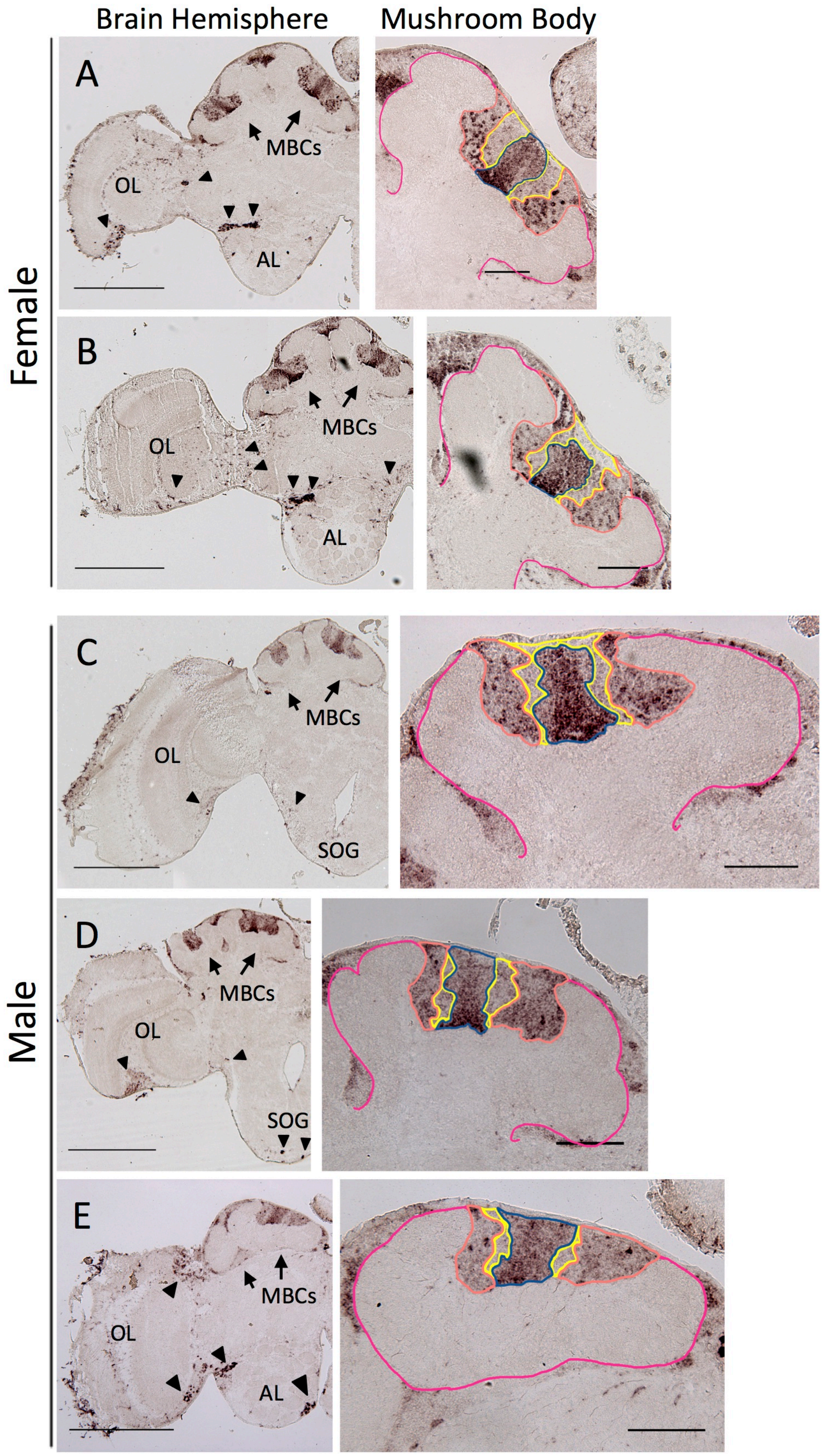

Supplementary Figure 1*. In situ* hybridization of *Trp* in hairy flower wasp *C. prismatica.*
(A-E) Adult brains of five individuals hybridized with antisense Trp probes. Left panels show frontal sections of the brain hemisphere. Trp signals scattered outside MBs are marked with arrowheads. Right panels show magnified views of the mushroom bodies shown in their left panels. Calyces are outlined with red lines. Cells were divided into three regions based on the location of somata within the calyces and Trp signal intensity. Inner core (strong Trp signal), middle (weak), inner peripheral (moderate) regions are encircled with blue, yellow and orange line, respectively. (A-B) Females, which are a different individual from the one shown in Fig. 2. (C-E) Males. MBC, mushroom body calyx; OL, optic lobe; SOG, suboesophageal ganglion; AL, antennal lobe. Bars indicate 500 µm in the left panels, 100 µm in the right panels.

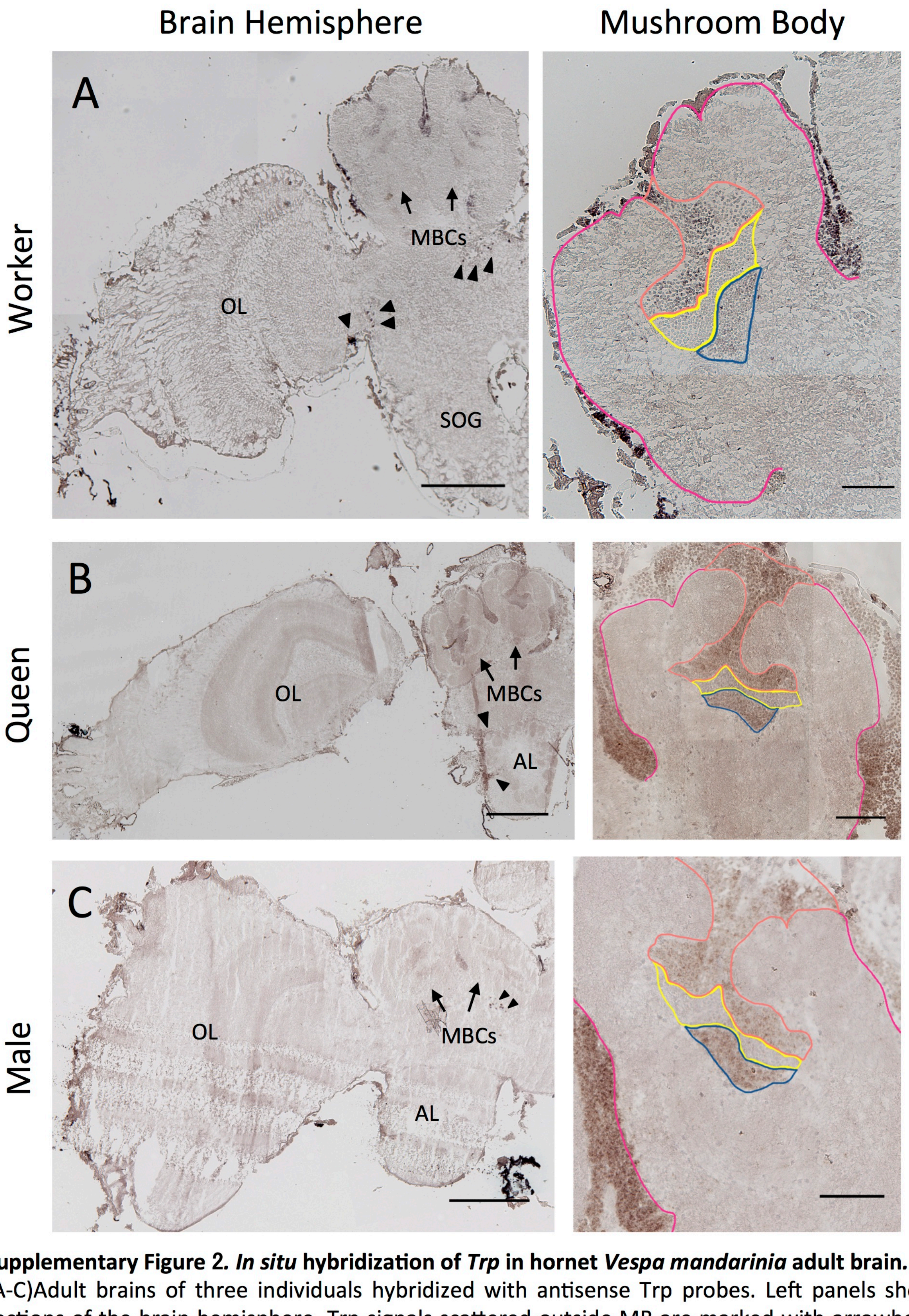

Supplementary Figure 2. ***In situ*** **hybridization of** ***Trp*** **in hornet** ***Vespa mandarinia*** **adult brain.**
(A-C)Adult brains of three individuals hybridized with antisense Trp probes. Left panels show frontal sections of the brain hemisphere. Trp signals scattered outside MB are marked with arrowheads. Right panels show magnified views of the mushroom bodies shown in their left panels (A,B) or of the serial section of the left panel (C). Calyces are outlined with red lines. Cells were divided into three regions based on the location of somata within the calyces and Trp signal intensity. Inner core (moderate Trp signal), middle (weak), inner peripheral (strong) regions are encircled with blue, yellow and orange line, respectively. (A) Worker, which is a different individual from the one shown in Fig. 3. (B) Queen. (C) Male. MBC, mushroom body calyx; OL, optic lobe; SOG, suboesophageal ganglion; AL, antennal lobe. Bars indicate 500 µm in the left panels, 100 µm in the right panels.

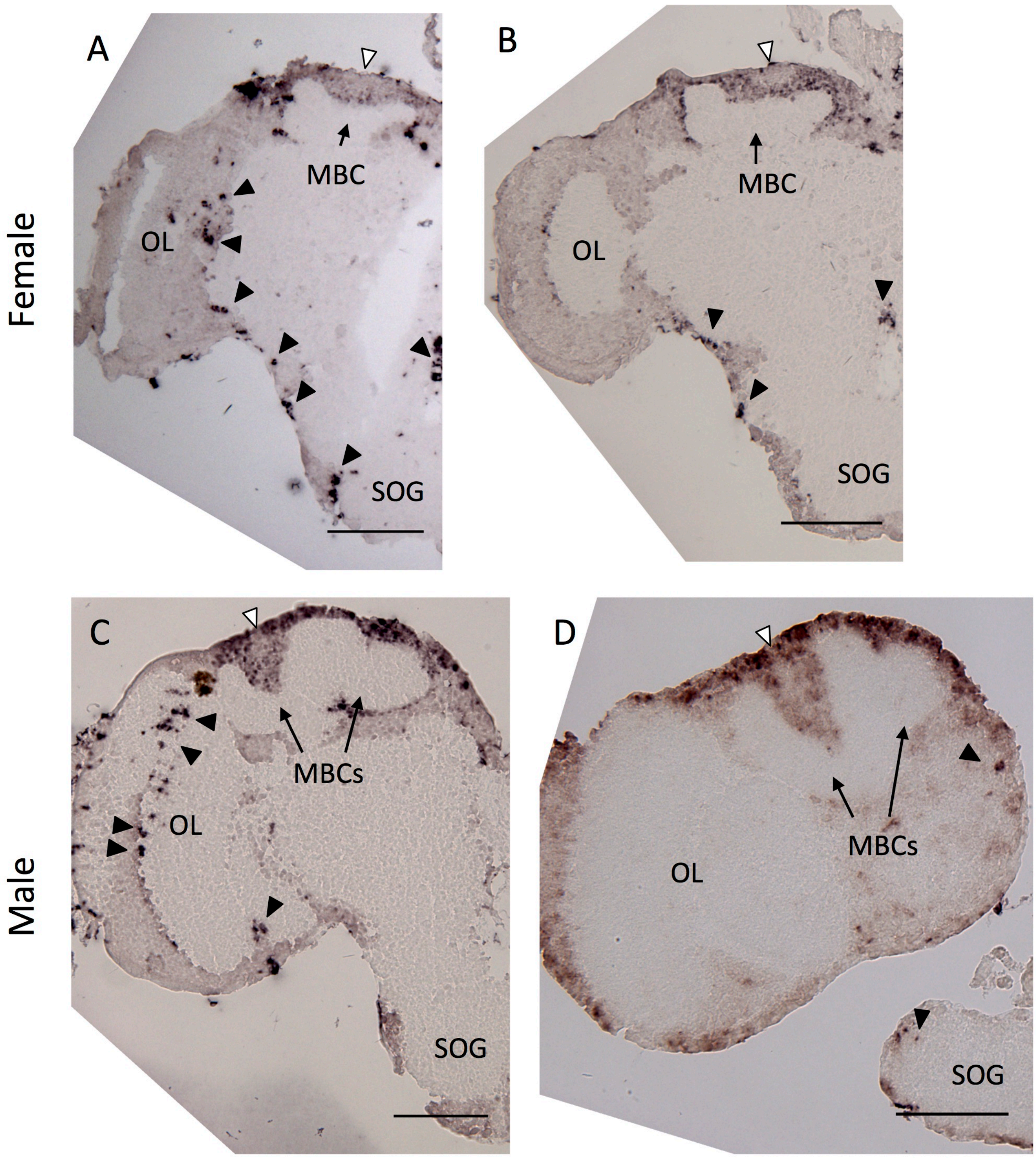

Supplementary Figure 3*. In situ* hybridization of *Trp in* parasitoid wasp *A. reticulata.*
(A-D) Frontal sections of the brain hemisphere of four individuals hybridized with antisense *Trp* probes. *Trp* signals scattered outside MBs are marked with black arrowheads. White arrowheads indicate the regions of Kenyon cells inside the calyx with relatively weak *Trp* signal. (A-B) Females, which are a different individuals from the one shown in Fig.4. (C-D) Males. MBC, mushroom body calyx; OL, optic lobe; SOG, suboesophageal ganglion. Bars indicate 100 μm.

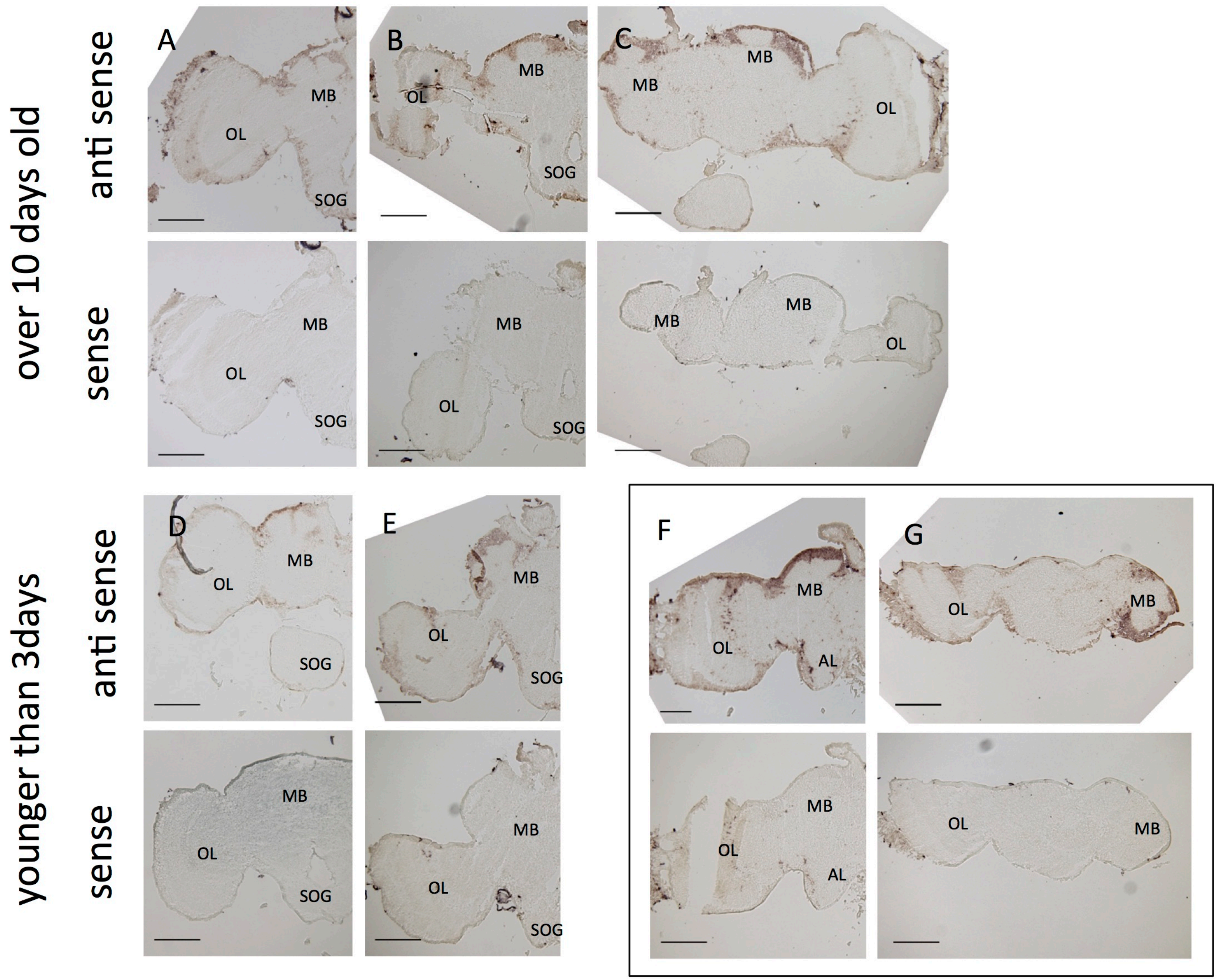

Supplementary Figure 4*. In situ* hybridization of *Trp* in sawfly *Arge similis* adult brain*.*
(A-C) Brains of adults older than 10 days after emergence. (D-G) Brains of adults younger than 3days after emergence. (A-E) show ubiquitous *Trp* expression like Fig. 5(C, D) while in (F,G) the expression is stronger in MBs than the other parts of brains, as in Fig. 5(A,B). MB, mushroom body; OL, optic lobe; SOG, suboesophageal ganglion; AL, antennal lobe. Bars indicate 200 µm.

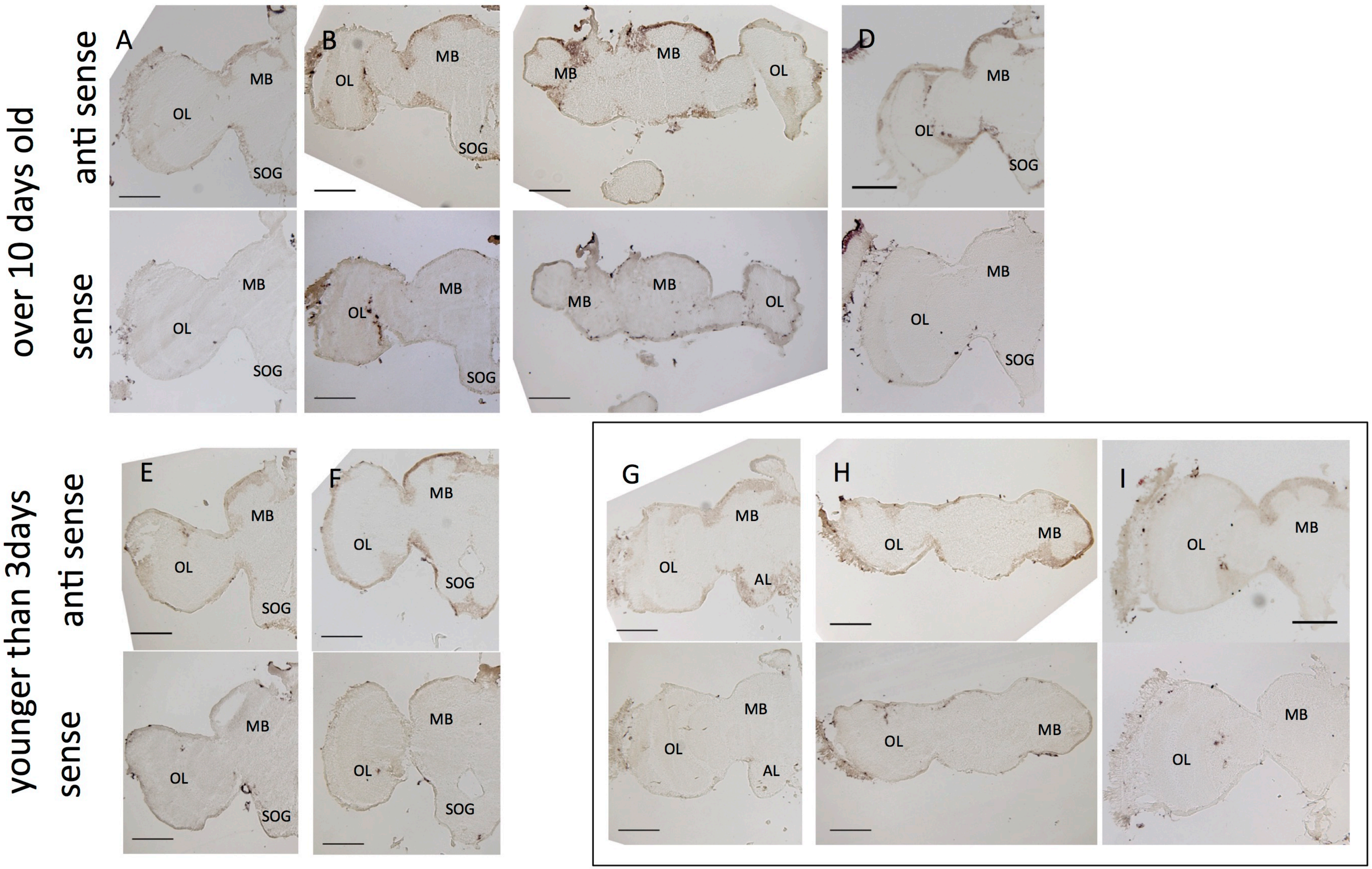

Supplementary Figure 5. ***In situ*** **hybridization of EF1** α **in sawfly** ***Arge similis*** **adult brain.**
(A-D) Brains of adults older than 10 days after emergence. (A-C) are serial sections of the same individuals as Supplementary Fig. 1 (A-C), (D) as Fig. 5 (C,D). (E-I) Brains of adults younger than 3days after emergence. (E-H) are serial sections of the same individuals Supplementary Fig. 1 (D-G), (I) as Fig. 5 (A,B). (A-F) show ubiquitous *Trp* expression (Sfig.4,Fig.5), while in (G-I) the expression is stronger in MBs than the other parts of brains(Sfig.4,Fig.5). MB, mushroom body; OL, optic lobe; SOG, suboesophageal ganglion; AL, antennal lobe. Bars indicate 200 µm.
